# Flexible Threshold-Type Switching Devices with Low Threshold and High Stability Based on Silkworm Hemolymph

**DOI:** 10.3390/nano12203709

**Published:** 2022-10-21

**Authors:** Lu Wang, Jing Yang, Hongyu Zhu, Wenhao Li, Dianzhong Wen

**Affiliations:** 1 School of Electronic Engineering, Heilongjiang University, Harbin 150080, China; 2 HLJ Province Key Laboratory of Senior-Education for Electronic Engineering, Heilongjiang University, Harbin 150080, China

**Keywords:** biononvolatile memory, silkworm hemolymph, graphene quantum dots, multifunctional, flexible

## Abstract

In this paper, a floating-gate flexible nonvolatile memory is reported that is composed of natural biological materials, namely, silkworm hemolymph, graphene quantum dots as the floating-gate layer, and polymethyl methacrylate (PMMA) as the insulating layer. The device has a high ON/OFF current ratio (4.76 × 10^6^), a low setting voltage (<−1.75 V), and good durability and retention ability. The device has two storage characteristics, namely, Flash and WORM, which can be effectively and accurately controlled by adjusting the limiting current during device setting. The resistance switching characteristics are the result of the formation and fracture of conductive filaments. The floating-gate flexible bioresistive random access memory prepared in this paper provides a new idea for the development of multifunctional and biocompatible flexible memory.

## 1. Introduction

With the development of science and technology, ever-changing electronic products are quickly updated, and scrapped devices produce a large amount of e-waste, which contains many difficult-to-degrade substances that are harmful to human health. To solve these problems, people have considered using natural biological materials to make electronic devices [1,2,3,4]. Electronic devices prepared by biomaterials are environmentally friendly, biocompatible, and sustainable [5,6,7,8], and the process of making devices using biomaterials is simple and does not require a large amount of energy consumption. According to previous reports, biomaterials have been successfully used in memristors, such as silk [2,9,10,11], egg white [1], lignin [12], and aloe vera [13,14]. Storage density, storage window, read–write speed, and data retention ability are important indicators of the performance of memristors. The storage characteristics of devices are improved through the improvement of device structure, material and the manufacturing process [15,16]. Ag/Ag-LGB/FTO memristors were fabricated by doping Ag nanoparticles with different mass fractions in Lophatherum gracile Brongn. The device doped with 2 wt% Ag nanoparticles had good stability, a maximum resistance window, and good storage performance [17]. An ITO/silk:Au-NP/Al memristor was fabricated by doping gold nanoparticles in silk fibroin protein. The current switching ratio of the device was improved to 10^6^, and the operating voltage was less than ±2 V [18]. A high-density silk fibroin memristor was fabricated with UV lithography. The size of the memristor was reduced to a few microns, and its storage density was 25 times higher than that of existing memristors [9]. Au/starch/ITO devices have the switching characteristic of a sudden change in resistance. After doping with chitosan, the Au/starch−chitosan/ITO device showed a slowly changing resistance switch behavior [19]. In ITO/GO/EA/GO/Al devices based on egg white and GO, the switch current ratio of the devices was adjusted through the GO concentration [20].

Flexible resistive memory has good flexibility, ductility, free-bending and folding, wearability, and other advantages [21,22,23], so it will occupy an important position in the future memory field. However, the dielectric layer materials of traditional memristors (such as bulk materials and nanoparticles) poorly adhere to the substrate and are easy to separate and crack in the bending state [24]. However, biomaterials are compatible with flexible substrates and have good flexibility and high mechanical strength, which brings a new idea for the development of flexible resistive memory. An MG/collagen/ITO/PET flexible memristor prepared by extracting collagen from fish skin showed basic synaptic behavior [25]. A cross-bar Ag/Au−chitosan/Au memristor fabricated on a transparent flexible substrate exhibited bipolar switching behavior [26]. A biological memristor based on silk protein and gold nanoparticles was successfully prepared on a flexible substrate with a switch ratio of 10^6^ [18].

Silkworm hemolymph is a natural biomaterial obtained from the silkworm body that contains abundant protein and can be used as the medium layer material of resistive memory. In this paper, based on the resistive properties of silkworm hemolymph (SH) material, the trap depth is deepened, and the ON/OFF current ratio is increased by inserting graphene quantum dots into the dielectric layer. A floating-gate resistive memory is fabricated by using SH and graphene quantum dot (GQD) composites as the floating-gate layer, and polymethyl methacrylate (PMMA) as the insulating layer. By adjusting the limited current and voltage scanning mode, the device can show flash and write-once-read-many (WORM) storage characteristics, and the set voltage is reduced. The device still maintains the resistance switching behavior in the bending state, and its resistance change characteristics remain unchanged after 10^4^ bending cycles, thus showing good mechanical properties and stability.

## 2. Materials and Methods

### 2.1. Materials and Device Fabrication

ITO/glass and ITO/PET (2 cm × 2 cm) were used as the bottom electrode and substrate materials, which were successively incubated with acetone, ethanol, and deionized water for ultrasonic cleaning for 30 min. The SH liquid was coated on the cleaned substrate at 1000 rpm for 20 s and dried at 80 °C for 1 h to obtain the dried active layer. Then, Al/SH/ITO/glass and Al/SH/ITO/PET devices were fabricated via thermal evaporation at a pressure of 2 × 10^−3^ Pa. GQDs (1 mg/mL) were purchased from Suzhou Tanfeng Graphene Technology Co., Ltd. (Suzhou, China). They had an average diameter of 15 nm, a thickness of 0.5 to 2.0 nm, and a purity of 80%. GQDs and SH solution were mixed at a volume ratio of 1:3, and the mixture was homogenized by ultrasonic stirring for 10 min. PMMA powder was dissolved in anisole to obtain a PMMA solution with a concentration of 70 mg/mL. The PMMA solution was coated on the cleaned substrate at a rate of 1000 rpm for 20 s and then vacuum-dried at 80 °C for 1 h. Then, the mixed solution of SH and GQDs was coated on the substrate at a speed of 1000 rpm for 20 s and vacuum-dried at 80 °C for 1 h. Then, another PMMA insulating layer was spin-coated on the dry dielectric layer by the same process and then dried. Finally, Al/PMMA/SH:GQD/PMMA/ITO/glass and Al/PMMA/SH:GQD/PMMA/ITO/PET devices were fabricated by evaporating the Al electrode at a pressure of 10^−3^ Pa.

### 2.2. Characterization

A scanning electron microscope (SEM, Hitachi S3400) (Hitachi, Tokyo, Japan) was used to observe the cross-section of the ITO–glass substrate coated with the SH film, and an energy-dispersive spectrometer (EDS, Model 550i, IXRF, Austin, TX, USA) was used to measure the elements in the film. Fourier transform infrared spectroscopy (FTIR, Thermo i410, NICOET, MA, USA) was used to measure the functional groups in the SH film. The electrical properties of the devices were measured by a semiconductor parameter tester (Keithley 4200, Keithley, Solo, OH, USA). Interventionary studies involving animals or humans and other studies that require ethical approval must list the authority that provided approval and the corresponding ethical approval code.

## 3. Results and Discussion

**Figure 1:**
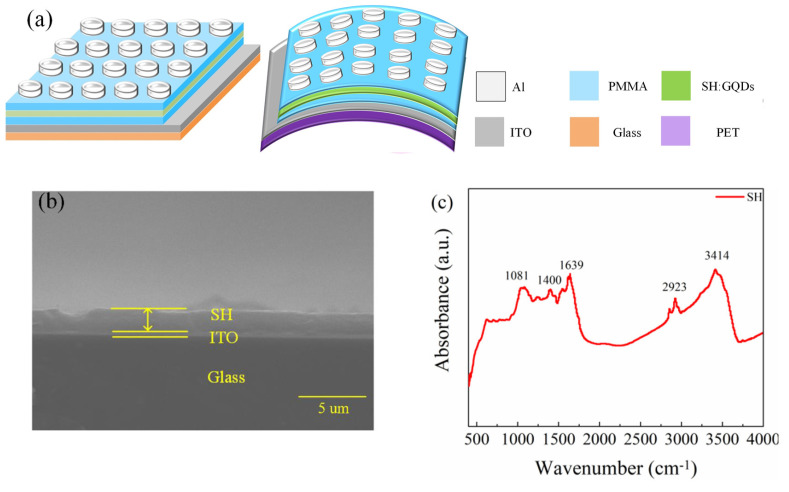
(**a**) Schematic diagram of the structure of floating-gate memristors. (**b**) SEM cross-section image of SH thin film. (**c**) Fourier infrared spectroscopy of SH thin films.

Figure 1a shows the schematic structure of Al/PMMA/SH:GQD/PMMA/ITO/glass and Al/PMMA/SH: GQD/PMMA/ITO/PET floating-gate devices, which consist of upper and lower electrodes, an insulation layer, a floating-gate, and a substrate. Scanning electron microscopy was used to observe the cross-section of the hemolymph protein membrane. Figure 1b shows the SH film, ITO electrode and glass substrate from top to bottom. The thickness of the SH film is approximately 2.02 µm. Fourier transform infrared spectroscopy (FTIR) was used to test SH, as shown in Figure 1c, where 1081 cm^−1^ corresponds to the C-C vibration of the protein ring structure. A small absorption peak appeared at 1400 cm^−1^, which is related to the composition of carboxylate. A relatively sharp peak appears at 1639 cm^−1^, corresponding to C=O. The relatively wide peaks related to -OH are in the range of 2600 cm^−1^~4000 cm^−1^ [13,27].

**Figure 2:**
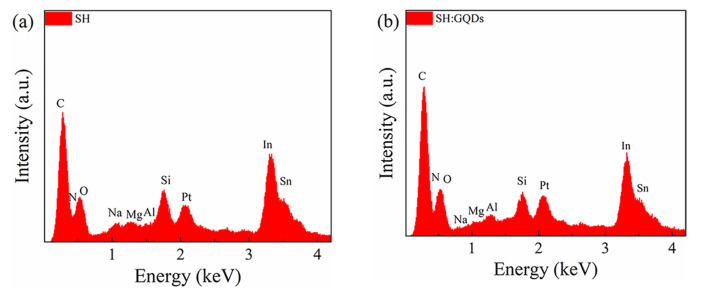
EDS analysis of the (**a**) SH thin film and (**b**) SH/GQD composite film.

To test the change in the composition of the dielectric layer caused by the addition of GQDs to the dielectric layer, the elements of the SH thin film and SH:GQD thin film were measured by EDS, as shown in Figure 2a,b. Si, In, Sn and Al come from the ITO glass substrate and electrode materials, and Pt comes from the gold spraying treatment during the test. SH contains C, N, O, Na, and Mg. The comparison between Figure 2a,b shows that the mass fraction of C and O in the composite increases significantly after introducing GQDs into SH, in which the mass fraction of C increases from 16.73 wt% to 20.31 wt%, and the mass fraction of O increases from 10.17 wt% to 10.63 wt%.

**Figure 3:**
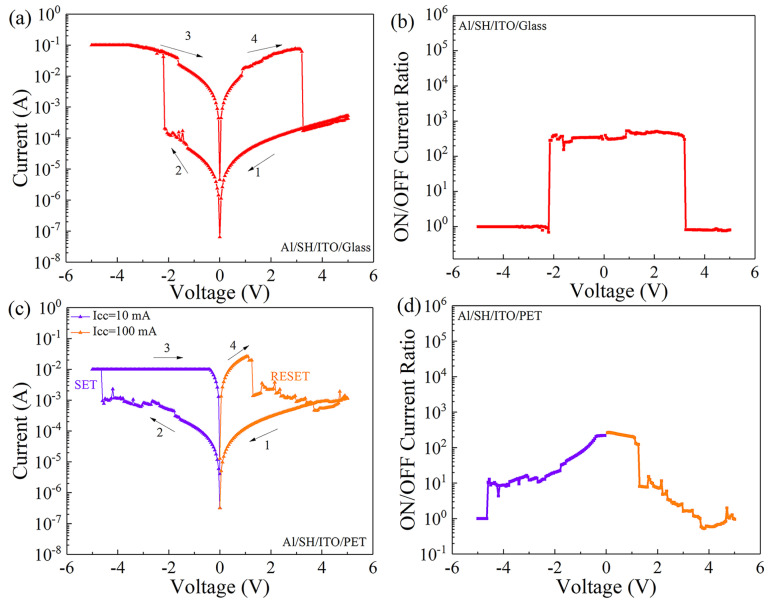
Al/SH/ITO/glass device: (**a**) I-V characteristic curve and (**b**) ON/OFF current ratio. Al/SH/ITO/PET device: (**c**) I-V characteristic curve and (**d**) ON/OFF current ratio.

Under DC voltage, the I-V characteristic curve of the Al/SH/ITO/glass device based on SH is tested, as shown in Figure 3a. In the test process, a DC scanning voltage was applied to the top electrode of Al, and the bottom electrode of ITO was grounded. The voltage scanning mode was 5 V→0 V→−5 V→0 V→5 V. When the negative DC voltage increases from 0 V to −2.15 V, the current of the device increases from 2.06 × 10^−4^ A to 5.99 × 10^−2^ A, indicating that the resistance state of the device changes from a high resistance state (HRS) to a low resistance state (LRS). The LRS remains stable from −5 V to 0 V. During the following voltage sweep from 0 V to 5 V, the current of the device decreases from 6.18 × 10^−2^ A to 1.72 × 10^−4^ A when the reset voltage exceeds 3.20 V, and the resistance state of the device reverts from LRS to HRS. Figure 3b shows the relationship between the ON/OFF current ratio of Al/SH/ITO/glass devices and the applied voltage. When the external applied voltage varies from −2.15 V to 3.20 V, the ON/OFF current ratio varies in the range of 10^2^~10^3^, and the maximum current switching ratio is 5.29 × 10^2^. The electrical characteristics of the Al/SH/ITO/PET devices were tested, as shown in Figure 3c. (I_CC_) refers to the imposed compliance current. I_CC_ = 100 mA was set in the positive voltage region, I_CC_ = 10 mA was set in the negative voltage region, and the voltage scanning direction was 0 V→−5 V→0 V→5 V→0 V. In the negative voltage region, when the voltage increases to −4.65 V, the current of the device suddenly increases, and the device switches from the HRS to the LRS. In the positive voltage region, when the voltage increases to 1.30 V, the current of the device suddenly decreases, and the device resumes HRS. Under continuous voltage scanning, the device can switch between the HRS and LRS. The ON/OFF current ratio of the device varies with the voltage, as shown in Figure 3d, and the maximum ON/OFF current ratio is 2.62 × 10^2^.

**Figure 4:**
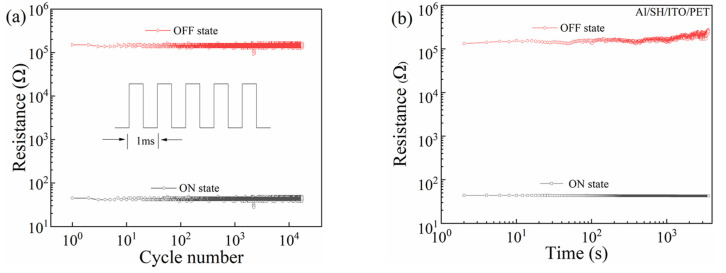
Al/SH/ITO/PET device: (**a**) pulse test and (**b**) retention time.

To investigate the influence of pulse voltage on the stability of Al/SH/ITO/PET devices, pulse tests were performed under a 1 V pulse. As shown in Figure 4a, the high and low resistance states of the devices remained stable for more than 10^4^ pulse cycles. The resistance of the high and low resistance states of the device at a 1 V DC voltage is shown in Figure 4b.

**Figure 5:**
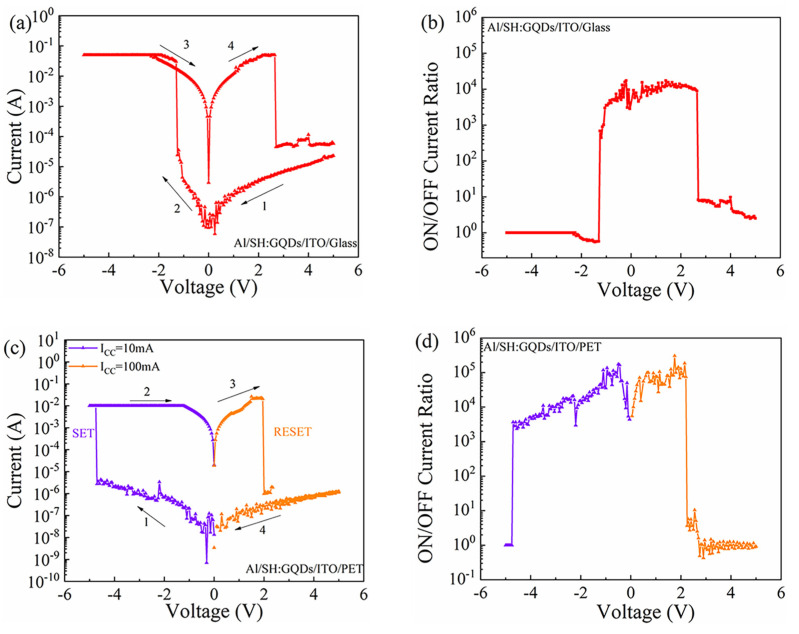
Al/SH:GQD/ITO/glass device: (**a**) I-V characteristic curve and (**b**) ON/OFF current ratio. Al/SH:GQDs/ITO/PET device: (**c**) I-V characteristic curve and (**d**) ON/OFF current ratio.

The I-V characteristic curve of the Al/SH:GQD/ITO/glass device under DC voltage scanning is shown in Figure 5a. The test results show that the Al/SH:GQDs/ITO/glass device has the characteristics of bipolar resistance switching. Due to the addition of GQDs in the dielectric layer, the current switching ratio of the device increases, and the set voltage and reset voltage are −1.25 V and 2.65 V, respectively. The current switch of the Al/SH:GQD/ITO/glass device is shown in Figure 5b. When the applied voltage changes from −1.25 V to 2.65 V, the ON/OFF current ratio varies within the range of 10^3^~10^5^, and the maximum ON/OFF current ratio is 1.71 × 10^4^. Introducing GQDs into the dielectric layer can increase the ON/OFF current ratio of the device, which helps to reduce the misreading probability of the device. The electrical characteristics of the Al/SH:GQD/ITO/PET devices were tested, as shown in Figure 5c. I_CC_ = 100 mA was set in the positive voltage region, I_CC_ = 10 mA was set in the negative voltage region, and the voltage scanning direction was 0 V→−5 V→0 V→5 V→0 V. In the negative voltage region, when the voltage increases to −4.75 V, the current of the device suddenly increases, and the device switches from the HRS to the LRS. In the positive voltage region, when the voltage increases to 1.95 V, the current of the device suddenly decreases, and the device resumes HRS. Under continuous voltage scanning, the device can switch between high and low resistance states. The ON/OFF current ratio of the device as a function of voltage is shown in Figure 5d, and the maximum ON/OFF current ratio of the device is 3.02 × 10^5^.

**Figure 6:**
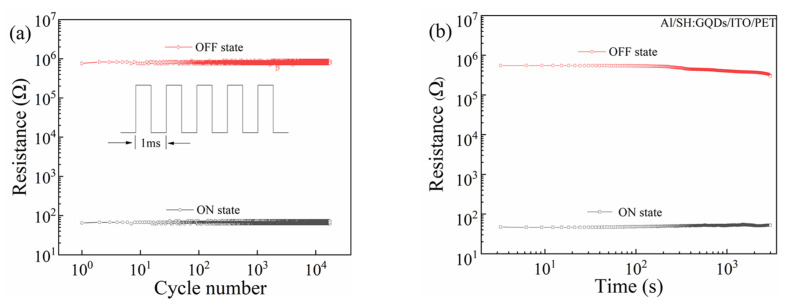
Al/SH:GQD/ITO/PET device: (**a**) pulse test and (**b**) retention time.

The effect of pulses on the stability of the Al/SH:GQD/ITO/PET device was tested under a 1 V pulse. As shown in Figure 6a, the high and low resistance states of the device remained stable for more than 104 cycles. The resistance value of the device under 1 V is shown in Figure 6b, and the retention time of the device exceeds 3 × 103 s.

**Figure 7:**
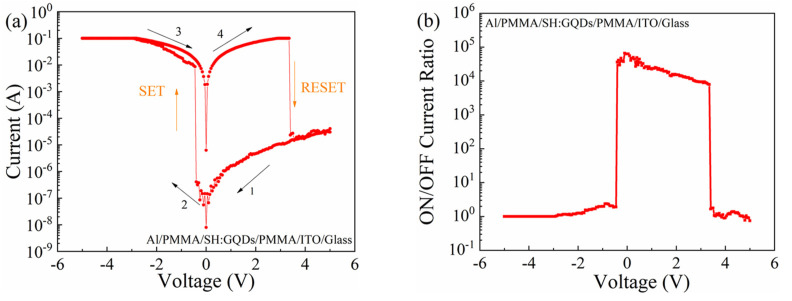
Al/PMMA/SH:GQD/PMMA/ITO/glass device: (**a**) I-V characteristic curve and (**b**) ON/OFF current ratio.

The I-V characteristic curve of the Al/PMMA/SH:GQD/PMMA/ITO/glass device is shown in Figure 7a, and the device shows bipolar resistance switching behavior. The initial state of the device is HRS; when the voltage is 5 V→0 V, the device maintains HRS. During the application of 0 V→−5 V to the device, when the voltage increases to −0.55 V (set voltage (Vset) = −0.55 V), the current suddenly increases from 1.35 × 10−5 A to 1.06 × 10−2 A, resulting in device switching from the HRS to the LRS. When a voltage of −5 V→0 V is applied to the device, the device remains in the LRS. During the voltage application of 0 V→5 V, when the voltage increases to 3.95 V (reset voltage (Vreset) = 3.95 V), the current suddenly decreases from 8.12 × 10^−2^ A to 8.13 × 10^−5^ A, causing the device to recover from LRS to HRS.

The ON/OFF current ratio versus voltage curve is shown in Figure 7b, and the maximum ON/OFF current ratio is 6.55 × 10^4^.

**Figure 8:**
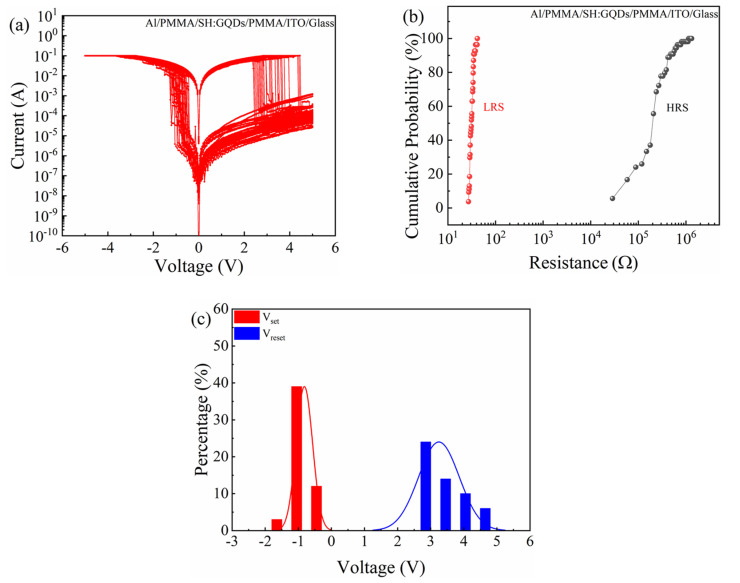
Al/PMMA/SH:GQDs/PMMA/ITO/glass device: (**a**) I-V characteristic curves of continuous voltage scanning. (**b**) Cumulative probability of resistance. (**c**) Distributions of V_set_ and V_reset_.

The Al/PMMA/SH:GQDs/PMMA/ITO/glass device can be switched continuously many times on the same memory cell, as shown in Figure 8a, which shows that the device has stable resistance switching behavior. To analyze the reliability of the device, the resistance of the high resistance state (R_HRS_) and resistance of the low resistance state (R_LRS_) at 1 V voltage were calculated from the I-V characteristic curve of continuous voltage scanning. Figure 8b shows the cumulative probability of R_HRS_ and R_LRS_. The median value of R_HRS_ is 2.20 × 10^5^ Ω, the median value of R_LRS_ is 31.99 Ω, and the coefficients of variation are 0.84 and 0.11. The coefficient of variation of R_LRS_ is smaller, and the resistance state is more stable. Figure 8c shows the histogram of the set voltage and reset voltage distribution of the Al/PMMA/SH:GQDs/PMMA/ITO/glass device in consecutive voltage scanning cycles. The average value of V_set_ is −0.82 V, the standard deviation is 0.24, and the coefficient of variation is 0.30. The average value of V_reset_ is 3.24 V, the standard deviation is 0.60, and the coefficient of variation is 0.19. The V_set_ distribution of the device is relatively stable.

**Figure 9:**
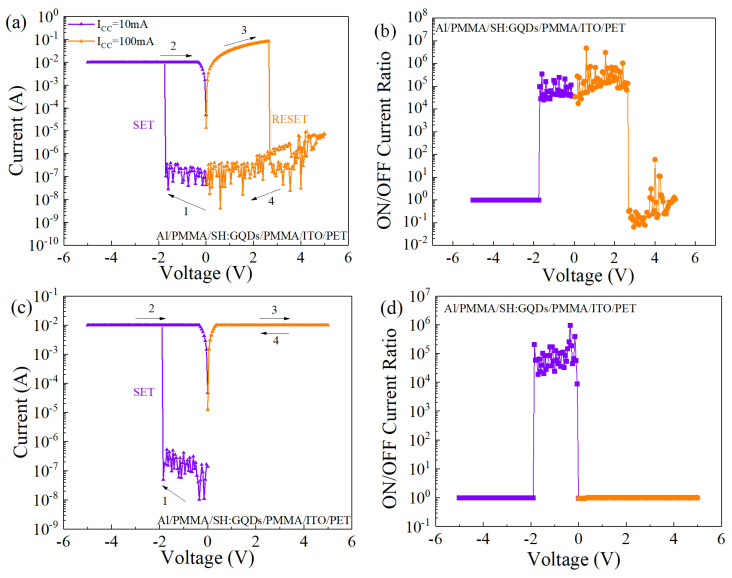
Electrical characteristics of the Al/PMMA/SH:GQDs/PMMA/ITO/PET device at different compliance currents: (**a**,**c**) I-V characteristic curve and (**b**,**d**) ON/OFF current ratio.

The Al/PMMA/SH:GQDs/PMMA/ITO/PET flexible device exhibits two different electrical characteristics under different compliance currents. As shown in Figure 9a, when a compliance current of 10 mA was set in the positive voltage region and a compliance current of 100 mA was set in the negative voltage region, the device exhibited bipolar switching characteristics. The ON/OFF current ratio of the device as a function of voltage is shown in Figure 9b, with a maximum ON/OFF current ratio of 4.76 × 10^6^. As shown in Figure 9c, a compliance current of 1 mA is set in the positive and negative voltage regions. When the positive voltage is −1.90 V, the current suddenly increases, and the device switches to LRS. During the subsequent positive and negative bias scanning, the device remained in the ON state, showing the storage behavior of WORM. As shown in Figure 9d, the maximum ON/OFF current ratio in the positive voltage region is 9.59 × 10^5^.

**Figure 10:**
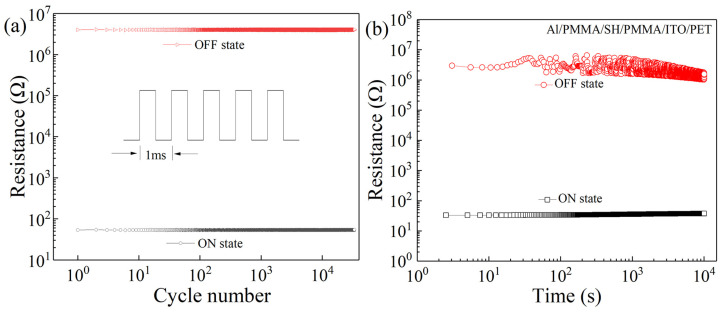
Al/PMMA/SH:GQD/PMMA/ITO/PET device: (**a**) pulse test and (**b**) retention time.

To study the influence of pulse voltage on the stability of the Al/PMMA/SH:GQD/PMMA/ITO/PET device, a pulse test was carried out under a 1 V pulse. As shown in Figure 10a, the high and low resistance states of the device can remain stable for more than 10^4^ cycles. The retention time of the Al/PMMA/SH:GQD/PMMA/ITO/PET device is shown in Figure 10b. The HRS and LRS can be stable within 10^4^ s.

**Figure 11:**
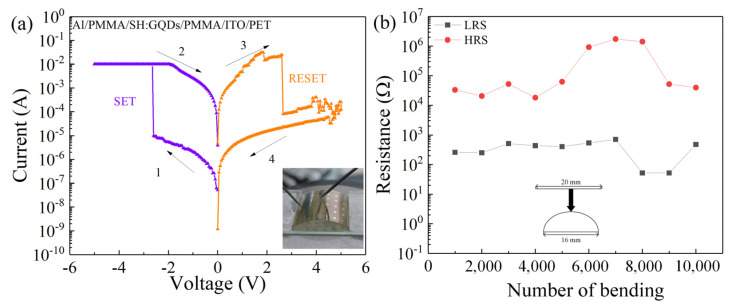
(**a**) I-V characteristic curve of the Al/PMMA/SH:GQD/PMMA/ITO/PET device in the bending state. (**b**) R_LRS_ and R_HRS_ after 10^4^ bending cycles.

The I-V characteristic curve of the device in the bending state is shown in Figure 11a, and the device still maintains the bipolar resistance switching behavior. In addition, to test its stability after bending, we tested whether the device could withstand the maximum curvature radius of 9.8 mm, curved 10^4^ times after high and low resistance state distribution, as shown in Figure 11b. According to the results before bending 5000 stable performance, high and low resistance state fluctuation is not substantial and continues to increase the bending times, high and low resistance states, and performance degradation.

**Figure 12:**
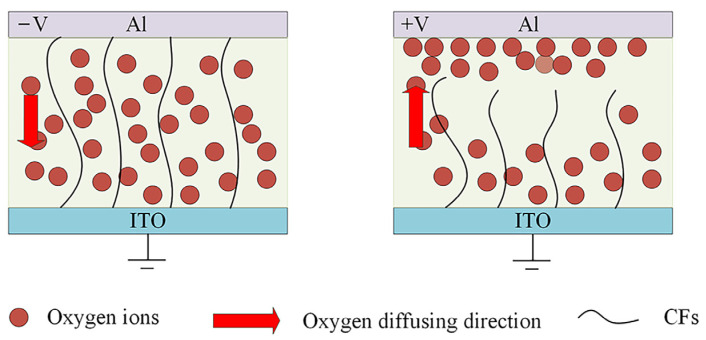
Conductivity diagram of the Al/SH/ITO device.

The oxygen functional groups in SH include carboxyl and hydroxyl groups, so we speculate that the formation and fracture of conductive filaments caused by the diffusion of oxygen ions is the main reason for the switching behavior of the device. The conductivity diagram of the Al/SH/ITO device is shown in Figure 12. Under the action of negative pressure, the negatively charged oxygen ions in SH diffuse along the electrode to form conductive filaments (CFs), and the device is in a state of low resistance. However, at positive voltages, oxygen ions move upward, the conductive wire breaks, and the storage device is in a high resistance state. The formation and fracture of conductive wires in Al/SH/ITO devices is the result of the movement of oxygen ions during setting and resetting.

**Figure 13:**
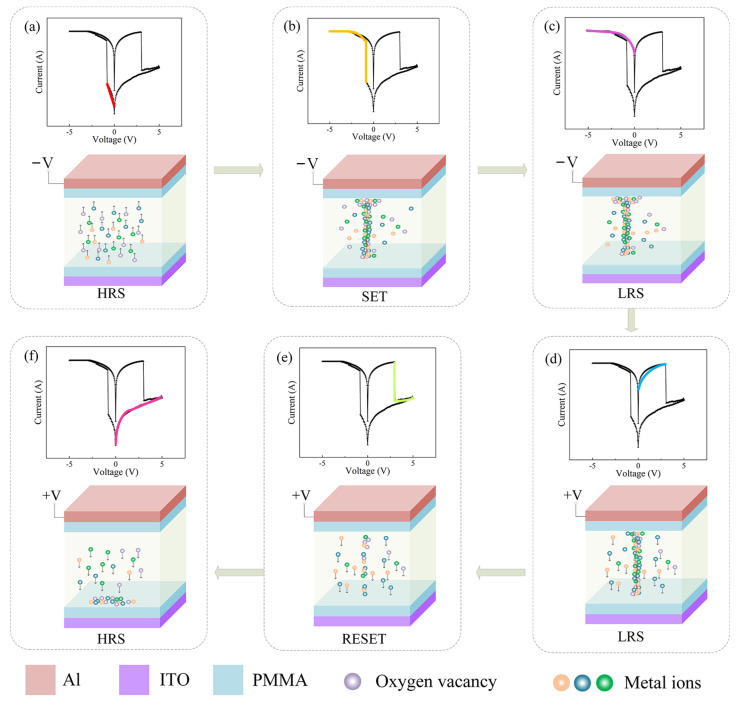
Conductivity diagram of the Al/PMMA/SH:GQD/PMMA/ITO device. (**a**–**f**) is divided into an upper and lower part: the upper part shows the IV curve of the device (different phases of the characteristic test are shown in different colors); the lower part shows the movement of oxygen vacancies and other metal ions in the dielectric layer during this phase.

The Oxygen functional groups in SH include carboxyl, hydroxyl and other metal ions, adding GQDs to the dielectric layer will introduce more traps [28,29,30], the conductive mechanism of Al/PMMA/SH:GQD/PMMA/ITO device is shown in Figure 13. Each figure in Figure 13a–f is divided into an upper and lower part. The upper part shows the IV curve of the device (different phases of the characteristic test are shown in different colors), and the lower part shows the movement of oxygen vacancies and other metal ions in the dielectric layer during this phase. First, in the top electrode where a negative voltage is applied, as shown in Figure 13a, the medium layer of oxygen vacancies and GQD oxygen vacancies and other metal ions in the electric field move up under the action. When the voltage continues to increase, as shown in Figure 13b, the oxygen vacancy and other metal ions, due to the effect of a high electric field in the dielectric layer formed in the conductive filament, turn into LRS [31,32]. Due to the existence of the PMMA insulation layer, it is difficult for electrons to move freely in the device [33], and the device will maintain the LRS, as shown in Figure 13c, if the negative voltage is continued. When a forward voltage is applied, electrons move downward, as shown in Figure 13d. When a sufficiently large forward voltage is applied, the conductive filaments dissolve, and the device recovers HRS, as shown in Figure 13e. Under the action of an electric field, oxygen vacancies and metal ions constantly move to the lower electrode, as shown in Figure 13f. Thus, the resistance state of the device can be repeatedly switched by applying a voltage to induce the formation and fracture of the conductive wires.

## 4. Conclusions

In this paper, Al/SH:GQD/ITO devices and Al/SH:GQD/ITO devices are fabricated based on the resistance characteristics of SH and the charge capture characteristics of GQDs. On the glass substrate, the switching current ratio increased from 5.29 × 10^2^ to 1.71 × 10^4^ after GQD incorporation, compared with SH. On the PET substrate, the switching current ratio increased from 2.62 × 10^2^ to 3.02 × 10^5^ after GQD incorporation, compared with SH. The switching current ratio of the two structures increased after GQD incorporation. To improve the structure of the device, the introduction of PMMA, which acts as an insulating layer, and the preparation of the Al/PMMA/SH:GQD/PMMA/ITO/glass floating-gate structure of biological resistance changing memory can switch more than 50 times on the same storage unit. The threshold voltage of the device is small and can be used in low power storage. The Al/PMMA/SH:GQD/PMMA/ITO/PET device under different current limitations and scanning voltages shows two kinds of electrical properties (flash and WORM), and the resistance time can be maintained for more than 10^4^ s. This work proves that floating-gate-type flexible biological resistance changing memory can play a flexible biological role in the field of electronics.

## Data Availability

The data presented in this study are available on request from the corresponding author.
